# Analyzing musculoskeletal system discomforts and risk factors in computer-using office workers

**DOI:** 10.12669/pjms.326.11436

**Published:** 2016

**Authors:** Melek Ardahan, Hatice Simsek

**Affiliations:** 1 Dr. Melek Ardahan, Department of Public Health Nursing, Ege University, Faculty of Nursing, İzmir, Turkey; 2 Hatice Simsek, Department of Public Health Nursing, Ege University, Faculty of Nursing, İzmir, Turkey

**Keywords:** Musculoskeletal discomfort, Computer-usage, Office workers

## Abstract

**Objective:**

This study analyzed the prevalence of work-related computer-user musculoskeletal discomforts, personal and computer-related risk factors.

**Methods:**

A cross-sectional survey on 395 office workers was made between July-September 2015. Musculoskeletal symptoms and risk factors were evaluated for participants’ demographics and job attributes on the 21-item questionnaire and the Turkish-Cornell Musculoskeletal Discomfort Questionnaire.

**Results:**

Participants reported musculoskeletal symptoms in the neck (67.85%), back (66.33%), lower back (59.49%), right shoulder (45.32%) and left shoulder (43.54%) during the past week and work interference was 33.6%, 28.5%, 30.6%, 31.3% and 31.9%, respectively. Musculoskeletal discomfort risks were being male, increasing daily computer usage, feeling computer-usage discomfort, hours working at desk and having knowledge about ergonomic exercises.

**Conclusion:**

Musculoskeletal symptoms are common in Turkish office workers and indicated the need for more attention to musculoskeletal disorders and designing effective preventive interventions.

## INTRODUCTION

Work-related musculoskeletal diseases (WMSDs) have been observed commonly and an increase in the frequency of symptoms through the years has been observed with the rapid development of computer technologies and ever-increasing usage of computers.^1^-^3^

Office workers are forming a very important part of the risk group for musculoskeletal system disorders as they are spending long working hours in front of a computer. Long-term usage of computers, working at a desk and sitting for a long time in a chair in workplaces are the main reasons playing a role in the musculoskeletal system disorders of office workers. In the literature, a relationship between computer usage and musculoskeletal system disorders was clearly observed. On this topic, it was found out that musculoskeletal disorders were common among office workers in Iran,^3^-^5^ Thailand,^6^,^7^ Estonia,^8^ India,^9^ Turkey,^1^,^10^-^12^ Malaysia^13^,^14^ and the United States.^2^

According to Britain Labour Force Survey (2015), a total of 9.5 million work days were lost as a result of the WMSD cases, which is a loss of 17 days per person. It was determined that 40% of the cases that resulted in a work-related time loss comprised of WMSDs.^15^ According to the report of the U.S. Bureau of Labor Statistics (2014), WMSDs formed 32% of all the injury and disease cases and the frequency of WMSD occurrence was found to be 33.8% per 10,000 full-time workers. Workers with WMSDs required an average of 13 days to get back to work.^16^ This study was planned for analyzing the frequency of musculoskeletal disorders and their risk factors among computer-using office workers.

## METHODS

The study included 490 government officials working at a government office in Manisa, Turkey. No sampling methods were applied. All the office workers who volunteered to participate in the study were included (n: 395). Participation rate in the study was 80.6%. Individuals with chronic pain related to a disease (cancer and neuropathic pain), diagnosed rheumatic disease and individuals who had received pain-related treatment within the last three months were excluded. Study data were collected between July-September 2015.

Data used in the study were collected in accordance with the Descriptive Attributes Form, which was created by scanning the literature and the “Cornell Musculoskeletal Discomfort Questionnaire” scale. The Turkish validity-reliability study was conducted by Erdinç et al. (2008)^17^ The T-CMDQ evaluates the frequency of occurrence of the situation, severity of the situation and whether or not it has affected the ability to do work during the last 7 days for 11 different body parts (neck, shoulders, back, upper arms, forearms, wrists, waist, hips, upper legs, knees and lower legs). On the questionnaire form, questions were asked on age, gender, educational level, marital status, height, weight, body mass index, chronic diseases, smoking, exercising, daily working hours at desk, daily working hours at computer, total years of computer use, status of physical or eye discomfort while using a computer, level of ergonomic knowledge and methods used for treating musculoskeletal system pains. Permission was obtained for conducting the study from the Ethical Committee of the Ege University, School of Nursing and permission was obtained with an organization application permit form from the Manisa Directorate of Tax Administration. Also verbal approvals were obtained from the office workers during the study.

The SPSS 15.0 software was used for statistical analysis. The Chi-square test and logistic regression analysis were used to validate the statistical significance between the possible risk factors and symptoms of the musculoskeletal system for computer users, while the Mann-Whitney U test was used for comparing the related risk factors with the scale total score. The results were evaluated at a 95% level of reliability and p<0.05 was evaluated as statistically significant.

## RESULTS

The average age of the office workers was 45.03±8.85 (min: 24, max: 65, median: 46 years) and 63.3% were males and 36.7% were females. Of the office workers, 79.4% were graduates with an associate/bachelor’s degree; 80.3% were married, while 30.6% smoked, 83.0% did not exercise regularly, 46.3% were overweight, 18% were obese and 77.5% of the participants did not have any knowledge about ergonomic exercises. The average working hours at a desk was 7.46±1.49 (median: 8 hours); 76.2% of the office workers were using desktop computers, their average computer using durations was 6.57±2.03 (median: 7 hours); average years of computer usage was 14.06±5.64 (median: 15 years). As for the evaluation of taking a rest while working at computers, 26.3% were found not to be taking any break and their average working hours at a computer without a break was found to be 3.42±2.30 (median: 3 hours).

Participants reported musculoskeletal symptoms most commonly in the neck (67.85%), upper back (66.33%), lower back (59.49%), right shoulder (45.32%) and left shoulder (43.54%) during the past week and work interference related to the discomfort in these regions was 33.6%, 28.5%, 30.6%, 31.3% and 31.9%, respectively. There was a statistically significant relationship between gender, years of computer usage, duration of daily computer usage, duration of uninterrupted computer usage, presence of physical discomforts, presence of ergonomic knowledge and the total weighted scores for office workers (p<0.05) (Table-I).

**Table-I:** Comparison of total weighted score with risk factors.

Risk Factors	Total Weighted Score		

	n	Mean (SD)	p*
*Gender*			
Female	145	106.21±125.91	0.000
Male	250	55.95±99.91	
*Age*			
<46	199	64.10±98.35	0.086
≥46	196	84.85±124.91	
*Computer usage year*			
<15 years	240	65.54±104.52	0.011
≥15 years	155	89.65±123.00	
*Daily Computer Usage Duration*			
<7 hours	179	61.22±105.69	0.004
≥7 hours	216	85.32±117.24	
*Non-Resting Computer Usage Duration*			
<3 h/day	249	59.90±93.22	0.001
≥3 h/day	146	99.11±136.55	
*Physical Discomfort*			
Yes	275	95.10±122.64	0.000
No	120	26.95±64.22	
*Ergonomic Knowledge*			
Yes	280	85.73±123.50	0.000
No	115	46.81±73.84	

* Mann-Whithney U Test.

Comparison of the risk factors and most frequent body parts for observing musculoskeletal system disorders has been given in Table-II. The most frequent body parts observed for musculoskeletal disorders have been identified as back, lower back, neck, left and right shoulders. It was found in the statistical analysis that being male, using a computer for more than seven hours per day and using a computer for three hours without taking any break could cause risks on the other four body parts, except for the waist area, while using a computer for more than 15 years could cause risks on the neck and left shoulder (p<0.05). The lack of physical presence of discomfort and ergonomics information were found to be a risk factor for five body regions (p<0.05). Age was identified as not having any risk related to the musculoskeletal system (p>0.05).

**Table-II:** Comparison of the risk factors with the most frequent body parts observed for muscoskeletal system disorder.

Risk Factors	Muscoskeletal system disorder within last week										
	n	Back		Lower back		Neck		Right shoulder		Left shoulder	
		Yes(%)	No(%)	Yes(%)	No(%)	Yes(%)	No(%)	Yes(%)	No (%)	Yes(%)	No(%)
Gender										
Female	145	115(79.3)	30(20.7)	94(64.8)	51(35.2)	119(82.1)	26(17.9)	81(55.9)	64(44.1)	80(55.2)	65(44.8)
Male	250	147(58.8)	103(41.2)	141(56.4)	109(43.6)	149(59.6)	101(40.4)	98(39.2)	152(60.8)	92(36.8)	158(63.2)
OR (95%Cl)*	2.0 (0.23-0.60)			0.7(0.46-1.07)		0.3(0.20-0.53)		0.5(0.34-0.77)		0.5(0.31-0.72)	
p**	0.000			0.062		0.000		0.001		0.000	
Age											
<46	199	131(65.8)	68(34.2)	117(58.8)	82(41.2)	135(67.8)	64(32.2)	86(43.2)	113(56.8)	80(40.2)	119(59.8)
≥46	196	131(66.8)	65(33.2)	118(60.2)	78(39.8)	133(67.9)	63(32.1)	93(47.4)	103(52.6)	92(46.9)	104(53.1)
OR (95%Cl)*		1.0(0.63-1.63)		1.1(0.69-1.66)		1.0(0.61-1.64)		1.1(0.74-1.75)		1.3(0.81-1.94)	
p**		0.458		0.427		0.541		0.228		0.106	
Computer usage year											
<15 years	240	154(64.2)	86(35.8)	144(60.0)	96(40.0)	155(64.6)	85(35.4)	105(43.8)	135(56.3)	93(38.8)	147(61.3)
≥15 years	155	108(69.7)	47(30.3)	91(58.7)	64(41.3)	113(72.9)	42(27.1)	74(47.7)	81(52.3)	79(51.0)	76(49.0)
OR (95%Cl)*		1.2(0.72-1.89)		0.9(0.57-1.35)		1.4(0.82-2.26)		1.1(0.69-1.62)		1.6(1.03-2.45)	
p**		0.153		0.440		0.052		0.250		0.011	
Daily Computer Usage Duration											
<7 hours	179	108(60.3)	71(39.7)	99(55.3)	80(44.7)	111(62.0)	68(38.0)	70(39.1)	109(60.9)	67(37.4)	112(62.6)
≥7 hours	216	154(71.3)	62(28.7)	136(63.0)	80(37.0)	157(72.7)	59(27.3)	109(50.5)	107(49.5)	105(48.6)	111(51.4)
OR (95%Cl)*		1.63(1.07-2.49)		1.37(0.92-2.06)		1.63(1.07-2.49)		1.59(1.06-2.37)		1.58(1.06-2.37)	
p**		0.014		0.075		0.016		0.015		0.016	
Non-Resting Computer Usage Duration											
<3 h/day	249	154(61.8)	95(38.2)	145(58.2)	104(41.8)	157(63.1)	92(36.9)	99(39.8)	150(60.2)	99(39.8)	150(60.2)
≥3 h/day	146	108(74.0)	38(26.0)	90(61.6)	56(38.4)	111(76.0)	35(24.0)	80(54.8)	66(45.2)	73(50.0)	73(50.0)
OR (95%Cl)*		1.4(0.87-2.38)		1.0(0.61-1.52)		1.4(0.84-2.41)		1.61(1.04-2.51)		1.2(0.75-1.83)	
p**		0.009		0.288		0.005		0.003		0.030	
Physical Discomfort											
Yes	275	216(78.5)	59(21.5)	187(68.0)	88(32.0)	226(82.2)	49(17.8)	146(53.1)	129(46.9)	143(52.0)	132(48.0)
No	120	46(38.3)	74(61.7)	48(40.0)	72(60.0)	42(35.0)	78(65.0)	33(27.5)	87(72.5)	29(24.2)	91(75.8)
OR (95%Cl)*		0.2(0.14-0.38)		0.4(0.24-0.65)		0.1(0.08-0.24)		0.4(0.26-0.73)		0.4(0.24-0.70)	
p**		0.000		0.000		0.000		0.000		0.000	
Ergonomic Knowledge											
Yes	280	201(71.8)	79(28.2)	181(64.6)	99(35.4)	201(71.8)	79(28.2)	141(50.4)	139(49.6)	134(47.9)	146(52.1)
No	115	61(53.0)	54(47.0)	54(47.0)	61(53.0)	67(58.3)	48(41.7)	38(33.0)	77(67.0)	38(33.0)	77(67.0)
OR (95%Cl)*		0.6(0.34-0.91)		0.6(0.36-0.92)		0.79(0.47-1.33)		0.6(0.34-0.89)		0.7(0.41-1.07)	
p**		0.000		0.001		0.007		0.001		0.005	

* Logistic regression,

** Chi-square test.

## DISCUSSION

It is known that computers, which have become a fundamental part of our daily lives, are causing musculoskeletal symptoms.^10^,^12^ In this study, participants reported musculoskeletal symptoms most commonly in the neck, upper back, lower back, right shoulder and left shoulder during the past week. The results are consistent with previously published studies.^2^,^3^,^6^,^10^,^14^ Besides these studies, in the other studies musculoskeletal disorders were observed on only one body part. These parts were reported to be hands^18^ and knees.^4^,^5^

It was found in the literature that being female is a risk factor for musculoskeletal system disorders.^6^,^8^,^10^,^13^,^19^ In some of the studies, it was found that females have more pain in their shoulders,^6^ lower back^13^ and neck^8^ compared to men. In the present study, it was found that the total weighted score for women was significantly higher compared to men. However, it was also found that being male was a risk for the other four body parts, except waist area, for musculoskeletal system disorders.

In the present study, it was observed that age was not a risk factor for 5 areas of statistical significance, while it was also observed that it was the same in the comparison made with the total weighted score. Likewise, Damanhuri et al. (2014) also reached the same conclusion as no significant relationship was presented in their study.^13^ There are a limited number of studies in the literature where age was examined as a risk factor. In the study by Erdinç (2011), it was stated that those with younger ages have a risk for back,^12^ while in some studies, it was stated that musculoskeletal system disorders might increase with increasing age.^5^,^20^

It was found in the present study that computer usage of more than 15 years creates risks on the neck and left shoulder area and the total weighted score was higher for individuals who had used computers for more than 15 years. As in the literature, it was found that musculoskeletal disorders occur more in persons who work at computers for many years.^6^,^12^,^18^,^21^

As in the literature, it was found that musculoskeletal disorders increase as the duration of daily computer usage increases.^3^,^11^,^18^,^19^ Çalık et al. (2013) found a statistically significant relationship between musculoskeletal system disorders and computer usage of more than four hours/day.^10^ Likewise, it was found that daily computer usage for more than 7 hours was a risk for 4 other areas, other than the waist, and was found to be statistically significant. Also, it was found that this situation was supported by the comparison made with the total point score.

Literature search showed that individuals having breaks while working at computers were experiencing less pain,^8^,^19^ while the individuals not having breaks were experiencing more pain.^22^ Likewise, we also found that individuals who did not have breaks while working at a computer were experiencing more pains in their backs, necks and shoulders, while their total weight score was statistically lower in a significant manner compared to those who had breaks.

We also observed that feeling physical pain and having knowledge of ergonomics was a risk factor for each of the five areas of musculoskeletal disorders, while this finding was supported by the comparison made of the total point scores. Study by Çalık et al, found that participants experiencing physical pain were observed to have pain symptoms on all parts of their bodies.^10^ It was also found that individuals without any ergonomic knowledge felt greater lower back pain,^13^ while individuals with ergonomic knowledge had significantly declining musculoskeletal system complaints.^2^ This result shows the importance of decreasing the discomforts of individuals in workplaces by making ergonomic regulations and training.

## CONCLUSION

As the findings from the study revealed, it was found that office workers most commonly felt pain in their necks, backs, waists and shoulders and it interfered with their work as a result of this pain. While gender, years of computer usage, duration of daily computer usage, uninterrupted computer usage, presence of physical pain and lack of ergonomic knowledge were found to be a risk for musculoskeletal system disorders. Sufficient resting possibilities, better working conditions and training in physical exercises are required for preventing these disorders.

### Limitations of the study

This study was conducted at a single organization. Some of the office workers were excluded. However, it is thought that this situation increases the reliability of the results, despite the decreasing number of participants.
